# The influence of subclinical mastitis on the protein composition and protease activities of raw milk from lactating Thai-crossbred dairy cows

**DOI:** 10.14202/vetworld.2023.1363-1368

**Published:** 2023-06-17

**Authors:** Attapol Tiantong, Sirichai Eardmusic, Pipat Arunvipas, Jai-Wei Lee, Wilasinee Inyawilert

**Affiliations:** 1 Faculty of Animal Sciences and Agricultural Technology, Silpakorn University, Phetchaburi IT Campus, Cha-Am, Phetchaburi, 76120, Thailand; 2 Department of Large Animal and Wildlife Clinical Sciences, Faculty of Veterinary Medicine, Kasetsart University, Kamphaeng Saen Campus, Kamphaeng Saen, Nakhon Pathom, 73140, Thailand; 3 Department of Tropical Agriculture and International Cooperation, National Pingtung University of Science and Technology, Pingtung, 91201, Taiwan; 4 Department of Agricultural Science, Faculty of Agriculture Natural Resources and Environment, Naresuan University, Phitsanulok, 65000, Thailand

**Keywords:** milk protein, protease activity, subclinical mastitis, Thai-crossbred dairy cows

## Abstract

**Background and Aim:**

Mastitis in dairy cattle is associated with a high rate of morbidity and death, which has major implications for milk production and quality. This study aimed to investigate the protein component and the activity of matrix metalloproteinase-2 (MMP-2) and -9 (MMP-9) in raw milk samples with different testing scores determined using the California mastitis test (CMT).

**Materials and Methods:**

Thirty cows were employed in the study, and milk from each quarter was tested for subclinical mastitis (SCM). According to the results of CMT, raw milk samples were classified into five categories: Healthy (score 0), trace (score T), weakly positive (score 1), distinctly positive (score 2), and strongly positive (score 3) for somatic cell count (SCC). The total milk protein was analyzed using the Bio-Rad protein assay, and the milk protein composition was determined using the sodium dodecyl sulfate-polyacrylamide gel electrophoresis technique. In addition, gelatin zymography was used to evaluate changes in proteolytic abilities.

**Results:**

Milk samples with CMT scores of 1 and 3 had the highest total milk protein levels (32.25 ± 12.60 g/L and 32.50 ± 7.67 g/L, respectively), while the samples from healthy cows (CMT score 0) were only 6.75 ± 1.64 g/L. Globulin and lactoferrin were significantly increased in samples with a CMT score of 3 compared with those with other CMT scores. The bovine serum albumin level in samples with a CMT score of 2 was significantly (p < 0.05) higher than those with other CMT scores. No significant differences in casein abundance were found among samples with different CMT scores. Results from analysis of proteolytic activities demonstrated that the level of MMP-9 in samples with a CMT score of 3 was significantly (p < 0.05) higher than those with other CMT scores.

**Conclusion:**

The protein content and gelatinolytic activity of milk were drastically altered by the number of SCC, mainly due to SCM.

## Introduction

Mastitis is the most economically significant disease affecting dairy herds due to its high prevalence, high medication costs, increased labor, loss of production and milk quality, and consequently early culling of animals. On dairy farms, this illness is the leading cause of antibiotic use. Antibiotic use may be linked to the development of antimicrobial-resistant bacteria that pose a public health risk, as evidenced by antimicrobial residues in milk and dairy products [1]. The leading risk factors for mastitis incidence include inappropriate farm management [2], the application of dry-cow treatment [3], cows with high parity [4], and cows through physiological changes and stresses [5].

Subclinical mastitis (SCM) is the most detrimental type of mastitis, with no noticeable changes in the appearance of milk and udder. However, reduced milk production, elevated somatic cell count (SCC) rise, the presence of pathogens in milk, and altered milk composition can be observed [6]. Although many animals can have SCM, lactating cows are more susceptible to substantial changes in their milk constituents [7, 8]. Early detection of health issues in the udder is critical for dairy farmers and veterinarians to ensure the health of the animals and the quality and production of the milk. Regular bacteriologic testing of quarter milk samples is time-consuming and hampered by financial considerations [9].

Somatic cell count is frequently used to monitor udder health and milk quality as an indicator of mastitis. Several studies have found that as SCC increases, the activity of milk proteolytic enzyme increases concomitantly. Immediately following the elevation of SCC in milk, the activity of proteolytic enzymes dramatically increases [10]. Automatic electronic counters and CMT are the most accurate and widely used methods for determining the amount of SCC [11]. During inflammation, mastitis releases several proteases and recruits polymorphonuclear neutrophils (PMNs) to migrate across the blood/milk barrier [12]. The mediators that are generated during inflammation, such as histamine, tumor necrosis factor-α, interferon-γ, and acute-phase proteins, enhance the permeability of the tight junctions of the mammary epithelium [13], leading to the influx of proteases into the mammary gland [14]. Numerous mastitis etiological factors are known to facilitate the secretion of proteases [15].

Polymorphonuclears recruited during mastitis are responsible, at least partially, for the proteolytic activity in milk from cows with SCM [16]. Matrix metalloproteinases (MMPs) are a class of calcium-dependent zinc-containing endopeptidases that participate in the normal remodeling process of the extracellular matrix (ECM) in many tissues [17]. As the initial line of defense during mastitis, PMNs are assisted by MMPs to migrate to the mammary ducts [18]. According to the classification of this protease family, there are five subfamilies. Membrane-type MMPs, stromelysins, collagenases, gelatinases, and a fifth group have not been well characterized [19]. The MMP enzyme family, which includes gelatinases A (MMP-2) and B (MMP-9), begins the breakdown of the native fibrillar components of the ECM of vertebrates. Extracellular matrix damage caused by significant mastitis etiologic agents is often healed, so that tissue structures and functions are recovered [20]. Matrix metalloproteinases are crucial for ongoing inflammation in cases of recurrent mastitis. Inhibition of these enzymes could be a potential therapeutic approach [21].

In Thailand, neither using CMT scores as an indicator of mastitis nor the changes in milk quality associated with SCM have been thoroughly studied. Therefore, this study aimed to evaluate the impact of CMT scores on diagnosing SCM and changes in protein composition and proteolytic enzymes in milk from lactating Thai-crossbred dairy cows.

## Materials and Methods

### Ethical approval

This experimental design and sampling protocol were approved by the Institutional Animal Care and Use Committee of Silpakorn University. The project approval was numbered 20/2564.

### Study period and location

The study was conducted from December 2021 to September 2022 at the Faculty of Animal Sciences and Agricultural Technology Farm, Silpakorn University Phetchaburi IT campus, Thailand.

### Animals and samples

The cows had various lactation phases, and the daily average milk output was <15 kg/day. Cows were kept in accessible stall barns during the trial and were given access to an *ad libitum* complete mixed feed designed to suit their nutritional needs. The cows were given post-milking teat cleaning after milking.

For the experimental sampling, 10 healthy controls and 20 cows with SCM had their quarters of milk sampled right before the morning milking. The teats were adequately cleaned before being dried with a single-use paper towel. According to the California mastitis tests (CMTs), the experimental design was separated into five groups: Score 0 = Healthy, Score T = Trace of mastitis, Score 1 = Weak positive, Score 2 = Distinct positive, and Score 3 = Strong positive, the CMT results of each quarter are presented in Table-1. A total of 10 mL of milk were taken in a sterile centrifuge tube after the CMT and first skimming by 400× *g* at 4°C for 20 min to recover the clear supernatant. Next, fat-free cell-free supernatant was stored in aliquots under −20°C until used for protein composition and protease activity assay.

**Table-1:** The CMT result of each quarter.

	CMT scores*					n

	0	T	+1	+2	+3	
The number of positive quarters	40	28	25	18	9	120
Percent (%)	33.3	23.3	20.8	15.0	7.5	

* Evaluated based on the degree of milk gelling and the prevalence of milk abnormalities

### Milk protein concentration

The total milk protein concentration was evaluated using a Bio-Rad protein assay with a dry-binding reagent (5000002, Bio-Rad Laboratories, Hercules, CA, USA) in a microplate format (SPECTRO-Star Nano, BMG Labtech GmbH, Ortenberg, Germany). The protocol was conducted as directed in the instruction book.

### Sodium dodecyl sulfate-polyacrylamide gel electrophoresis (SDS-PAGE)

A 10% separating gel with the Laemmli sample buffer was used. Before conducting SDS-PAGE, the equivalent of 10 g of protein from the skim milk supernatant was combined with a 2X Native Sample Buffer (161–0738, Bio-Rad Laboratories). The staining process took place for 60 min with Coomassie blue dyes (161–0786, Bio-Rad Laboratories). Later, the samples were preserved in distilled water for band visibility. The probable protein components were found on air-dried gels, and the predicted band sizes for the proteins were 110–150 kDa for globulin, 70–80 kDa for lactoferrin, 50–60 kDa for bovine serum albumin (BSA), and 25–30 kDa for casein. The corresponding band pictures were recorded utilizing. The relevant band pictures were acquired using a CanoScan LiDE 120 Scanner (Canon Inc., Tokyo, Japan), and ImageJ (National Institute of Mental Health, Bethesda, Maryland, USA) was used to quantify them [22].

### Gelatin zymography

A total of 20 g of protein was present in an aliquot of the skimmed milk, which was then combined (1:1) with a sample buffer at pH 6.8 made up of 62.5 mM Tris-HCl, 25% glycerol, 4% SDS, and 0.01% bromophenol blue (Bio-Rad). After that, it was run through a 7.5% native SDS-PAGE (Minigel, Bio-Rad, California, USA) with 0.1% bovine gelatin (Sigma-Aldrich, Saint Louis, MO, USA) in the resolving gel. The gels were then incubated at 37°C for an overnight period in a 50 mM Tris-base buffer (Bio-Rad, California, USA) with pH 7.4 and 200 mM NaCl, 0.02% Brij-35, and 5 mM CaCl2 for gelatinolysis after 30 min at room temperature (25°C) in a renaturing solution of 2.5% (v/v) Triton X-100. The gels were stained for 30 min with 0.5% Coomassie Blue R-250 (Sigma-Aldrich) in 40% methanol, 10% glacial acetic acid, and 50% distilled water the following day. Next, they were destained for 30 min in 30% methanol, 7.5% glacial acetic acid, and 62.5% distilled water. Later, they were preserved in distilled water. Matrix metalloproteinase-2 and -9 were visible bands against the blue backdrop. After scanning with a CanoScan LiDE 120 Scanner (Canon Inc., Tokyo, Japan), it was integrated using ImageJ software version 1.80 (National Institute of Mental Health, Bethesda, Maryland, USA) [22]. The area of the band was used to represent the degree of gelatinase.

### Statistical analysis

The total protein, proteinous protein, and gelatinase activity concentrations were compared between the treatments according to the CMT results. A Duncan multiple range test was used to determine whether significant changes between treatments were made using R version 4.1.2 (R Core Team, 2021) [23]. All findings are presented as the mean ± the standard error. At p < 0.05, statistical significance was evaluated.

## Results

### Total milk protein content

**Figure-1:**
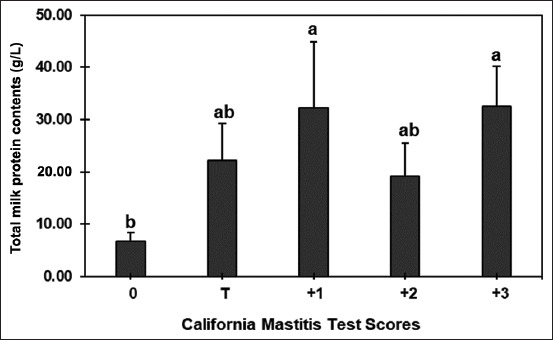
The total protein content of raw milk at various California mastitis test scores of Thai-crossbred dairy cows. ^a,b^Columns with different superscripts differ significantly (p < 0.05) among the multiple treatments.

The highest levels of total milk protein were identified in the milk sample at CMT scores 1 and 3 (32.25 ± 12.60 g/L and 32.50 ± 7.67 g/L, respectively), with a substantial increase when compared with the milk sample at CMT score 0 (6.75 ± 1.64 g/L). The total protein content of CMT outcomes with scores T and 2 did not differ significantly (22.17 ± 7.01 g/L and 19.25 ± 6.28 g/L, respectively). The results are shown in Figure-1.

### Protein composition of raw milk

**Figure-2:**
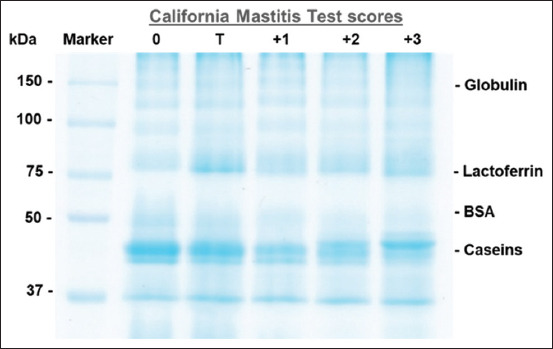
Images of globulin, lactoferrin, BSA, and casein bands in the raw milk of Thai-crossbred dairy cows with varying CMT scores.

**Figure-3:**
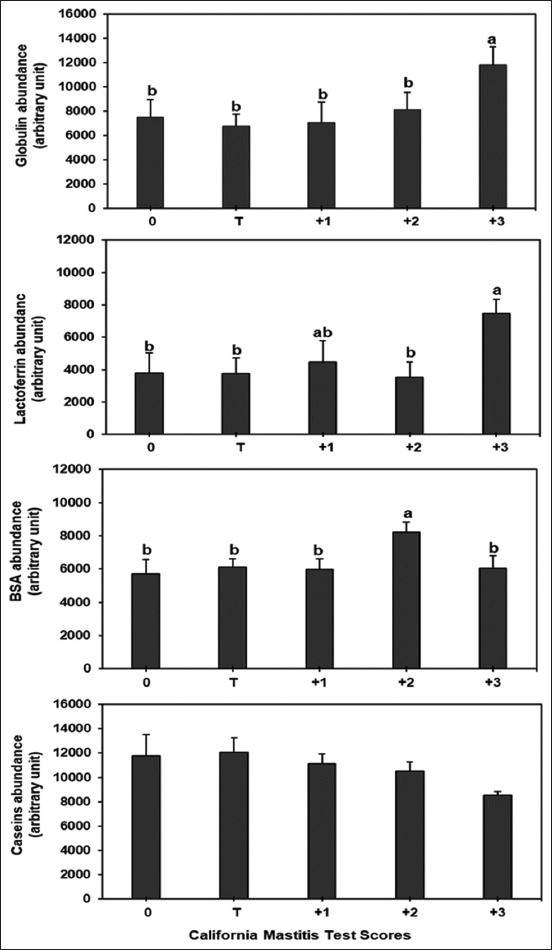
The abundance of globulin, lactoferrin, bovine serum albumin, and caseins of raw milk from Thai-crossbred dairy cows with varying California mastitis test scores. ^a,b^Columns with different superscripts differ significantly (p < 0.05) among the various treatments.

The hypothesized globulin, lactoferrin, BSA, and casein bands of the raw milk at the various CMT scores are shown in Figure-2. The abundance of globulin, lactoferrin, BSA, and casein (band area/g protein) in raw milk at the various CMT scores of Thai-crossbred dairy cows is depicted in Figure-3.

The abundance of globulin and lactoferrin in the raw milk with a CMT score of 3 increased significantly (p < 0.05), while other CMT scores were not significantly different. The abundance of BSA in raw milk increased (p < 0.05) at CMT score 2 compared with other CMT scores. We discovered no significant variation in casein abundance between the CMT scores.

### Matrix metalloproteinase-2 and MMP-9

**Figure-4:**
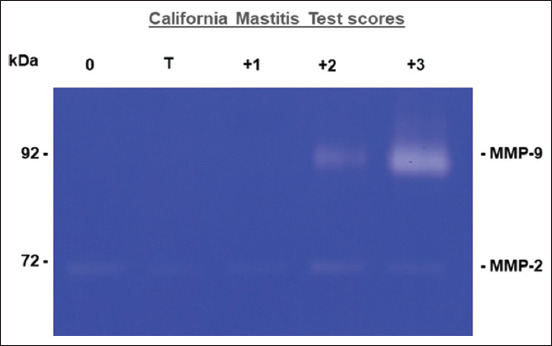
Image of matrix metalloproteinase (MMP)-2 and MMP-9 bands in the raw milk of Thai-crossbred dairy with varying California mastitis test scores.

**Figure-5:**
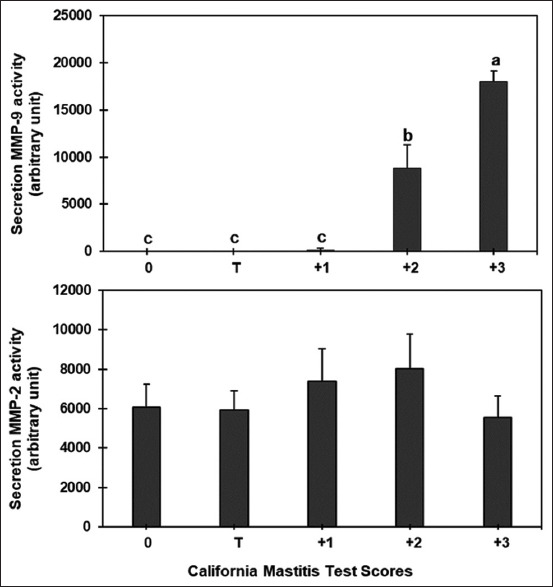
The gelatinolytic capacity level of raw milk at the various California mastitis test scores of Thai-crossbred dairy cows. ^a,b,c^Columns with different superscripts differ significantly (p < 0.05) among the multiple treatments.

The gelatin zymogram of the raw milk supernatant is shown in Figure-4, along with the different CMT scores. A visual representation of the incremental alterations that occurred in the mean band areas of 92 kDa (MMP-9) and 72 kDa (MMP-2) is shown in Figure-5. There was evidence of MMP-2 and MMP-9 in the zymogram for each CMT score.

The MMP-9 levels in the milk sample with a CMT score of 3 were significantly higher than the MMP-9 levels (p < 0.05) in the milk sample with a CMT score of 2, 1, T, and 0, respectively. The MMP-9 level in the milk sample with a CMT score of 2 was significantly higher than the MMP-9 level in the milk sample with a CMT score of 1, T, and 0 (Figure-5). The MMP-2 levels in the raw milk samples were not significantly different among the various CMT scores (Figure-5).

## Discussion

Mastitis directly impacts dairy farm profitability due to rejected milk, productivity, quality losses, and treatment expenditures [24]. This study examined the evolution of milk quality and the expression of crucial proteases in the milk of cows in Western Thailand. The study demonstrated a substantial correlation between high CMT scores and enhanced proteolytic activity.

According to research by Harmon [25], mammary gland inflammation can cause several compositional shifts in milk. These shifts can occur either as a result of local effects or as a result of serum components entering the milk and the movement of some normal milk components out of the alveolar lumen and into the perivascular space. In theory, any changes in the mammary secretion that occur during inflammation might be used to quantify the consequences of mastitis. However, issues with equipment and standardization have made it difficult for farms to use most tests.

The findings here showed that the abundance of total milk protein concentrations varied in proportion to CMT scores. These results are consistent with prior investigations, which documented several protein responses to mammary gland infection [26]. The pathogenesis of mastitis includes a complicated series of interactions between an invading pathogen and the immune system of the host. Considering the complexity and high dynamic range of the milk proteome, a quantitative proteomic method and related bioinformatics analysis are viewed as complementing tools for thoroughly investigating the dynamic interactions between the immune system and pathogens. Turk *et al*. [27] have demonstrated that milk protein is significantly altered during bovine mastitis.

The milk alterations in the previous experiment by Thomas *et al*. [28] are similar to the changes in the abundance of majoring whey protein identified in this investigation. Differences in protein abundance between subclinical and clinical mastitis have been studied earlier by Maity *et al*. [26]. This study revealed that the amount of globulin, lactoferrin, and BSA rose much higher in individuals with high CMT scores than in those with low CMT scores. Still, there was no considerable difference between the mild, moderate, and severe cases of SCM that were all positive. Globulin is the predominant isotype in the milk of ruminants. It is regarded as the primary opsonin facilitating neutrophil phagocytosis in the milk of infected mammary glands due to particular Fc receptors on neutrophils and macrophages in bovines that bind to globulin [29]. It is claimed that the high concentration of globulin may explain its crucial function in udder infection resistance.

Lactoferrin is a multifunctional glycoprotein in milk and other exocrine secretions that bind iron. Lactoferrin in milk is an inherent resistance element to prevent mammary gland infection by microbes [30]. An increase in the lactoferrin concentration in mastitis milk was reported by Niero *et al*. [31]. Numerous studies have clarified the defense mechanisms against udder infection. For instance, some of these defense mechanisms include the action of the complement system in infected mammary tissues, the mechanism of the onset of inflammation, the behavior of immune cells, the effects of lactoferrin on the immune system [32], the control of the production of several cytokines [33], and the inactivation of bacterial endotoxin and lipopolysaccharides [34]. In this study, we confirmed the results in which the lactoferrin concentration increased with high CMT scores.

The presence of BSA in colostrum, regular milk, and mastitis milk is well known. Inflammation increases capillary permeability, raising albumin milk concentration [10]. It is hypothesized that the BSA content in milk is a helpful indicator of the degree of inflammation in clinical mastitis [35]. We discovered slightly different results when comparing BSA concentration and CMT scores: The BSA concentration of CMT score 2 was higher than the CMT Score 3, differing from the results of the globulin and lactoferrin concentrations. This difference might need to be checked in a future study with more accurate methods, such as enzyme-linked immunosorbent assay.

Matrix metalloproteinases are physiologically significant mediators of the remodeling and destruction of almost all components of the ECM and basement membrane [10, 36]. The high CMT score levels in the current study increased the extracellular gelatinase levels in milk. Only gelatinase-type MMPs, identified as MMP-9, were responsible for this increase, whereas there were no differences in MMP-2 activity between the groups. The elevated gelatinase level harmed the basement membrane and interstitial tissues, culminating in the final loss of blood and inflammatory site barriers. This was associated with a rise in milk levels of small molecular weight plasma protein, globulin lactoferrin, and BSA. The elevation of MMP-9 in endotoxin-induced mastitis milk was also confirmed in naturally occurring diseases [37].

## Conclusion

Bovine mastitis is an infection of the mammary gland that produces major alterations in the protein content and gelatinolytic activity of milk. Identifying protein patterns in bovine milk is a specific and valuable technique for developing possible diagnostic biomarkers for mastitis and disease surveillance. Changes in milk abundance patterns help understand mastitis pathophysiology and serve as a valuable resource in developing innovative diagnostics.

## Authors’ Contributions

AT: Sample collection, investigation, statistical analysis, and writing original draft. SE, PA, and WI: Coordinated the research, data analysis, and manuscript preparation. JL: Supervision and writing-review, and editing. All authors have read, reviewed, and approved the final manuscript.
